# The key pathways and genes related to oncolytic Newcastle disease virus-induced phenotypic changes in ovarian cancer cells

**DOI:** 10.71150/jm.2411018

**Published:** 2025-04-29

**Authors:** Wei Song, Yuan Yuan, Fangfang Cao, Huazheng Pan, Yaqing Liu

**Affiliations:** Department of Clinical Laboratory, The Affiliated Hospital of Qingdao University, Qingdao 266000, P. R. China

**Keywords:** Newcastle disease virus, oncolytic virus, ovarian cancer, virotherapy

## Abstract

The poor prognosis and high recurrence rate of ovarian cancer highlight the urgent need to develop new therapeutic strategies. Oncolytic Newcastle disease virus (NDV) can kill cancer cells directly and regulate innate and adaptive immunity. In this study, ovarian cancer cells infected with or without velogenic NDV-BJ were subjected to a CCK-8 assay for detecting cell proliferation, flow cytometry for detecting the cell cycle and apoptosis, and wound healing and transwell assays for detecting cell migration and invasion. Transcriptomic sequencing was conducted to identify the differentially expressed genes (DEGs). GO and KEGG enrichment analyses were performed to explore the mechanism underlying the oncolytic effect of NDV on ovarian cancer cells. The results showed that infection with NDV inhibited ovarian cancer cell proliferation, migration, and invasion; disrupted the cell cycle; and promoted apoptosis. Compared with those in negative control cells, the numbers of upregulated and downregulated genes in ovarian cancer cells infected with NDV were 1,499 and 2,260, respectively. Thirteen KEGG pathways related to cell growth and death, cell mobility, and signal transduction were significantly enriched. Among these pathways, 48 DEGs, especially SESN2, HLA B/C/E, GADD45B, and RELA, that may be involved in the oncolytic process were screened, and qPCR analysis verified the reliability of the transcription data. This study discovered some key pathways and genes related to oncolytic NDV-induced phenotypic changes in ovarian cancer cells, which will guide our future research directions and help further explore the specific mechanisms by which infection with NDV suppresses ovarian cancer development.

## Introduction

Globally, ovarian cancer is the eighth most common cancer in women but the leading cause of death among all malignancies affecting the female reproductive tract (Webb and Jordan, 2024). According to the latest data from the National Cancer Institute, the estimated case fatality rate of ovarian cancer is 64.74% (Siegel et al., 2024). Ovarian cancer is an aggressive disease that is frequently detected at advanced stages, causing ovarian cancer to become the most fatal female reproductive cancer (Konstantinopoulos and Matulonis, 2023). The traditional treatment for ovarian cancer is a combination of cytoreductive surgery and systemic treatment with platinum-based chemotherapy. Unfortunately, 70% of patients with advanced ovarian cancer relapse, after which survival is extremely low (Nebgen et al., 2019). Patients often succumb to recurrence with chemotherapeutic resistance. Additionally, the immunotherapy response rates among ovarian cancer patients remain modest, mainly due to the immunosuppressive tumor microenvironment (TME) (Yang et al., 2020). The high recurrence rate, high case fatality rate, and severe chemoresistance highlight the urgent need to develop new therapeutic strategies.

Oncolytic virotherapy is an innovative and promising method for cancer treatment. Oncolytic viruses (OVs), including wild-type oncolytic viruses and genetically engineered viruses, are defined as agents that can selectively kill cancer cells while discriminating against healthy cells (Schirrmacher, 2022). Newcastle disease virus (NDV) is an inherent OV, and the permissive hosts of NDV are birds (Schirrmacher, 2016). In addition, the intravenous injection of NDV in nonhuman primates demonstrates the safety of NDV (Buijs et al., 2014).

NDV can directly kill human tumor cells. The structural proteins of NDV play crucial roles in damaging and inhibiting cancer cells. The concerted actions of the membrane proteins fusion (F) and haemagglutinin-neuraminidase (HN) make the virus membrane fuse with the cell membrane, causing NDV to enter host cells successfully (Liu et al., 2021) and eventually leading to cell death via apoptosis (Liang et al., 2021) and other death codes. Oncolytic virus anticancer therapy can be used not only as an independent treatment strategy but also as an adjustment for immunotherapy. NDV can activate both innate and adaptive immunity and regulate the tumor TME, causing it to transition from immunosuppressed to immunoactive, which will help augment immunotherapy response rates among ovarian cancer patients (Schirrmacher, 2022).

Since NDV was identified as an antineoplastic agent in 1965, it has been actively used to treat various tumors (Cassel and Garrett, 1965). A standardized NDV oncolysate vaccine was established in melanoma cell lines and applied in phase Ⅱ clinical trials with considerable results (Murray et al., 1977). A similar phase Ⅱ study of NDV oncolysates was conducted, involving 208 patients with locally advanced renal cell carcinoma, with a conclusion of improved disease-free survival (DFS) in comparison with the survival data of similar patients who were treated by surgery alone (Kirchner et al., 1995). An attenuated oncolytic NDV confers a significant benefit in the treatment of lung cancer (Yaacov et al., 2008). The autologous tumor cell vaccine ATV-NDV has been developed and applied in many clinical trials including patients with colorectal carcinoma (Bohle et al., 1990), breast cancer (Ahlert et al., 1997), head and neck squamous cell carcinomas (Karcher et al., 2004), renal carcinoma (Pomer et al., 1995), and glioblastoma multiforme (Steiner et al., 2004).

However, the promising oncolytic NDV is less commonly used to treat ovarian cancer. Several related studies have been conducted: a clinical trial revealed the clinical effectiveness of ATV-NDV vaccine in ovarian cancer (Ahlert et al., 1997); persistent infection was established in a human ovarian cancer cell line with a recombinant low-pathogenic NDV (Rangaswamy et al., 2017); combining 3TSR and NDV(F3aa) led to enhanced virus delivery and trafficking of immunologic cells into the primary tumor core (Matuszewska et al., 2019). Considering the severity of ovarian cancer and the efficiency and availability of oncolytic NDV, more studies of the utilization of NDV in the treatment of ovarian cancer are urgently needed. In this study, human ovarian cancer cell lines (SK-OV-3 and A2780) were infected with a velogenic NDV strain (NDV-BJ) to determine the influence of NDV on the proliferation, cell cycle, apoptosis, migration, and invasion of ovarian cancer cells. Afterward, transcriptomic sequencing and analysis were performed to explore the possible molecular mechanism underlying the oncolytic effect of NDV on ovarian cancer cells and identify potential key host factors, which can provide clues for further research on combining molecular therapy, oncolytic virotherapy, and immunotherapy in the treatment of ovarian cancer.

## Materials and Methods

### Cells and virus

The human ovarian cancer lines SK-OV-3 and A2780 and the human normal ovarian epithelial cell line IOSE-80 were obtained from IMMOCELL (China). Baby Hamster Syrian Kidney BHK-21 cells were purchased from Procell (China). They were cultured in Dulbecco’s modified Eagle’s medium (DMEM) (SK-OV-3 and A2780), RPMI 1640 medium (IOSE-80) or minimum essential medium (MEM) (BHK-21) (Procell, China) supplemented with 10% fetal bovine serum (FBS) (BI, Israel) and 1% penicillin-streptomycin mixture (Procell, China). All the cells were routinely incubated at 37℃ in a humidified atmosphere containing 5% CO_2_.

The velogenic NDV-BJ with mean death time (MDT) of 52.4 h and intracerebral pathogenicity index (ICPI) value of 1.95 was used in this study (Liu et al., 2019). Velogenic NDV strains have the potential to be developed as direct anticancer agents because of their multicyclic replication capacity (Feng et al., 2011). NDV was generated via inoculation into the allantoic cavity of 9-day-old chicken embryos. The collected allantoic fluid containing NDV was centrifuged at low speeds to remove coarse debris and at high speeds to concentrate and purify the virus in gradient sucrose solution. The virus liquid was subsequently passed through a 0.45 µm membrane filter and stored at -80℃. The titer of NDV was determined in BHK-21 cells via plaque assay.

### Viral infection

IOSE-80, SK-OV-3, and A2780 cells were seeded in 96-well plates. The next day, the cells were infected in triplicate with the indicated viruses at various multiplicities of infection (MOIs; 0, 0.005, 0.05, 0.5, 1, and 3). The cytopathic effect (CPE) was observed under an inverted microscope (Leica, Germany), and images were captured at 96 h post infection (hpi).

### Multistep viral growth assay

The cells were seeded in 96-well plates and infected with NDV at an MOI of 0.05 in basal DMEM or RPMI 1640 medium for an hour at 37℃. The virus liquid was removed and replaced with DMEM or RPMI 1640 medium supplemented with 2% FBS. At different time points (0, 24, 48, 72, and 96 hpi), the cell supernatant was collected for titration in BHK-21 cells via plaque assay. The cells in the plate were fixed with methanol for 10 min, blocked with PBS containing 3% bovine serum albumin (BSA) in PBS for 30 min and incubated at room temperature with anti-NDV serum (ab34402, Abcam, UK) as the primary antibody for 2 h and with Alexa Fluor 488-conjugated goat anti-chicken IgY (H + L) (ab150169, Abcam, UK) as the secondary antibody for one hour. Images of the bound fluorescence were captured with a fluorescence inverted microscope (Leica, Germany).

### Cell proliferation assay

Cell proliferation assay was detected by using the Cell Count Kit-8 (CCK-8) (Topscience, China) according to the manufacturer’s instructions. Cells were seeded in 96-well plates at a density of 2 × 10^4^ cells/well for IOSE-80 and 1 × 10^4^ cells/well for SK-OV-3 and A2780 and infected with NDV as described for the multistep viral growth assay. Next, CCK-8 solution (10 µl in 100 µl DMEM or RPMI 1640 medium) was added to each well at different time points (0, 24, 48, 72, and 96 hpi) and incubated at 37℃ for one hour. The optical density (OD) was recorded at a wavelength of 450 nm via a microplate reader (Bio-Rad, USA).

### Flow cytometric analysis of the cell cycle and apoptosis

Flow cytometry was performed to analyze the cell cycle and apoptosis. For the cell cycle, cells plated in 6-well plates and infected with or without NDV at an MOI of 0.05 were collected at 24 hpi and fixed with 80% (v/v) ethanol overnight at -20℃. The following processes were carried out according to the manufacturer’s instructions for the Cell Cycle Assay Kit (elabscience, China). Briefly, the cells were incubated with RNase A reagent at 37℃ for 30 min and stained with propidium iodide (PI) at 4℃ in the dark for 30 min.

For apoptosis, cells plated in 12-well plates and infected with or without NDV at an MOI of 0.05 were collected at 24 hpi. The following processes were carried out according to the manufacturer’s instructions for the Annexin V-FITC PI Apoptosis Kit (elabscience, China). Cells resuspended in Annexin V binding buffer were stained with Annexin V-FITC reagent and PI reagent at room temperature in the dark for 15 min. Cell cycle and apoptosis data were acquired via an Attune NxT flow cytometer (Thermo Fisher Scientific, USA) and analyzed via FlowJo software (version 10.8.1).

### Cell migration and invasion assays

A wound healing assay was carried out to detect cell migration. The cells were seeded in 6-well plates at a density of 2.5 × 10^6^ cells/well. When the confluence reached 100%, the cell monolayer was scratched with a 200 µl pipette tip to create a wound, which was infected with NDV at an MOI of 0.05. Wound healing over 48 h was monitored at certain time intervals. Images were taken from five different perspectives per well and analyzed via ImageJ software (version 1.52) to measure the wound area. The percentage of wound closure was quantified via the following equation: wound closure% = [1-(wound area at T_t_/wound area at T_0_)] ×100%.

The transwell assay was performed to assess cell migration and invasion. A total of 3 × 10^4^ cells in 100 µl serum-free medium were plated in 24-well uncoated inserts (Corning, USA) for migration assays or in Matrix gel-coated (Beyotime, China) inserts for invasion assays. First, 100 µl NDV liquid with an MOI of 0.05 or serum-free medium was added to the inserts. 500 µl medium supplemented with 10% FBS was added to the lower chamber as a chemoattractant. At 24 hpi, the cells in the upper chamber were completely removed with a wet cotton swab. The cells that passed through the inserts were fixed with 4% paraformaldehyde and stained with 0.1% crystal violet. The number of migrated or invaded cells in three random fields was quantified via an inverted microscope (Leica, Germany).

### Transcriptome sequencing and bioinformatic analysis

Transcriptome sequencing was performed by LC-Bio Technology Co., Ltd. Total RNA was extracted from cells infected with or without NDV at an MOI of 0.05 for 48 h with TRIzol reagent (Thermo Fisher Scientific, USA). The cDNA libraries were constructed with SuperScript^TM^ Ⅱ Reverse Transcriptase (Invitrogen, USA). The 2 × 150 bp paired-end sequencing (PE150) was performed on an Illumina NovaSeq^TM^ 6000 following the vendor's recommended protocol. To obtain high-quality clean reads, the reads were further filtered via Cutadapt software (version 1.9). HISAT2 software (version: hisat2-2.0.4) was used to align the reads to the reference genome. The mapped reads of each sample were assembled via StringTie (version 2.1.6) with default parameters. All the transcriptomes from all the samples were subsequently merged to reconstruct a comprehensive transcriptome via gffcompare software (version 0.9.8). After the final transcriptome was generated, StringTie and Ballgown were used to estimate the expression levels of all the transcripts, and the expression abundance of the mRNAs was determined by calculating the fragment per kilobase of transcript per million mapped reads (FPKM) value. The differentially expressed genes (DEGs) were identified via DESeq2 software on the basis of a |log_2_-fold change (FC)|> 1 and an FDR<0.05. The DEGs were subsequently subjected to GO (http://www.geneontology.org/) and KEGG (http://www.kegg.jp) enrichment analyses. The gene-pathway network was visualized via Cytoscape software (version 3.10.1). The protein-protein interaction (PPI) network was obtained via STRING (https://www.cn.string-db.org/) and visualized via Cytoscape software (version 3.10.1).

### RNA extraction and qPCR

The accuracy of the transcriptome sequencing was confirmed via qPCR analysis. The cells were infected with or without NDV at an MOI of 0.05. At 48 hpi, total RNA was extracted via RNAiso Easy (TaKaRa, Japan) according to the manufacturer’s instructions. Total purified RNA was reverse transcribed with random primers via the PrimeScript^TM^ FAST RT Reagent Kit with gDNA Eraser (TaKaRa, Japan). With the resulting cDNA used as a template for qPCR, real-time PCR with TB Green^®^ Premix Ex Taq^TM^ Ⅱ FAST qPCR (TaKaRa, Japan) was performed to detect the mRNA levels of the indicated genes. The relative mRNA levels of each target gene were quantified according to the 2^-ΔΔCt^ method, utilizing GAPDH as the reference gene. The primers used for qPCR are listed in Table S1.

### Western blot analysis

The cells were lysed in RIPA lysis buffer (Beyotime, China) for 10 min at 4℃. The supernatant was collected by centrifugation at 15000 r/min for 15 min at 4℃. After measuring protein concentration by the BCA Protein Assay Kit (Solarbio, Beijing, China), 20 μg protein samples were boiled, separated by 12.5% SDS-PAGE, and transferred to PVDF membrane (Millipore, USA). The membrane was blocked with 5% skim milk for one hour and then incubated overnight at 4℃ with antibodies against GADD45B (1:1000, Immunoway, China) and GAPDH (1:20000; #60004-1-Ig, Proteintech, China).

### Statistical analysis

Data were acquired from at least three independent experiments. Student’s *t*-test (two-tailed) was used to compare the data of two groups. Analysis of variance for three or more groups was determined using one-way ANOVA. The statistical analysis was performed with GraphPad Prism software (version 6.01). *P* < 0.05 was considered significantly different.

## Results

### NDV can grow quickly in ovarian cancer cells and suppress cell proliferation

**Fig. 1.**
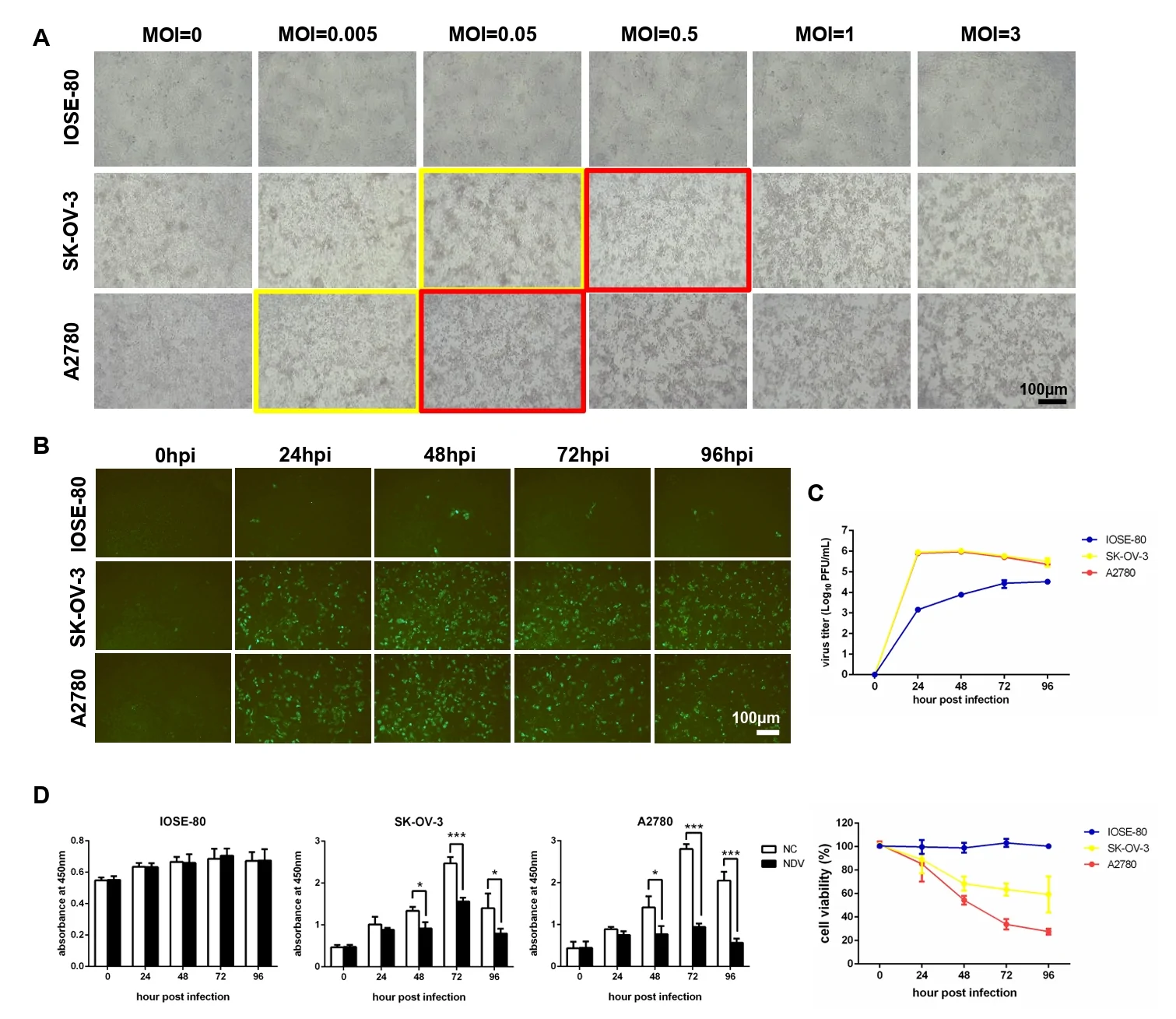
NDV can grow speedily in ovarian cancer cells and suppress cell proliferation. (A) Determination of the optimal MOI of NDV by observing the CPE. The yellow rectangles indicate the onset of CPE and the red rectangles indicate the obvious CPE. The multi-step growth of NDV was detected by IFA (B) and plaque assay (C). (D) The cell proliferation was detected by CCK-8 assay (^*^, *P* < 0.05; ^***^, *P* < 0.001; otherwise, *P* > 0.05).

To determine the optimal MOI of NDV, cells were infected with different MOIs (0, 0.005, 0.05, 0.5, 1, and 3) for 96 h. As shown in Fig. 1A, CPE occurred in A2780 cells when the MOI was 0.005. In SK-OV-3 cells, the MOI at which CPE occurred was 0.05. At this MOI, the CPE was more obvious in A2780 cells: the cells were wrinkled, rounded, and shed, and the cell count was reduced under a microscope. The in-control normal ovarian epithelial IOSE-80 cells grew well even at high MOIs. Overall, the virus was used for infection at an optimal MOI of 0.05 throughout this study.

Cells plated in 96-well plates were used to detect the virus via IFA and plaque assays. The virus could be stained with fluorescence from 24 to 96 hpi in SK-OV-3 and A2780 cells but was rarely detected in IOSE-80 cells. The fluorescence gradually weakened at later time points, likely because the cells were severely disrupted by NDV and shed extensively (Fig. 1B). The titer of the virus in the ovarian cancer cell lines SK-OV-3 and A2780 showed similar values and trends: the peak reached approximately 10^6^ at 48 hpi, and the maximum difference between ovarian cancer cells and normal ovarian cells was approximately 1,000 times. The titer in ovarian cancer cells decreased after 48 hpi; however, the titer in normal ovarian cells continuously increased (Fig. 1C).

To evaluate cell proliferation, a CCK-8 experiment was carried out, and the absorbance at 450 nm was obtained at different time points. As shown in Fig. 1D, the absorbance significantly decreased in the NDV-infected SK-OV-3 and A2780 cells at 48, 72, and 96 hpi compared with that in the negative control (NC) cells. The relative cell viability of the NDV-infected SK-OV-3 and A2780 cells at 96 hpi decreased to 59.22% and 27.51% that of the NC cells, respectively. The absorbance of the NDV-treated IOSE-80 cells even exceeded that of the NC cells, with no significant difference.

### Infection with NDV caused a disordered cell cycle and promoted the apoptosis of ovarian cancer cells

**Fig. 2.**
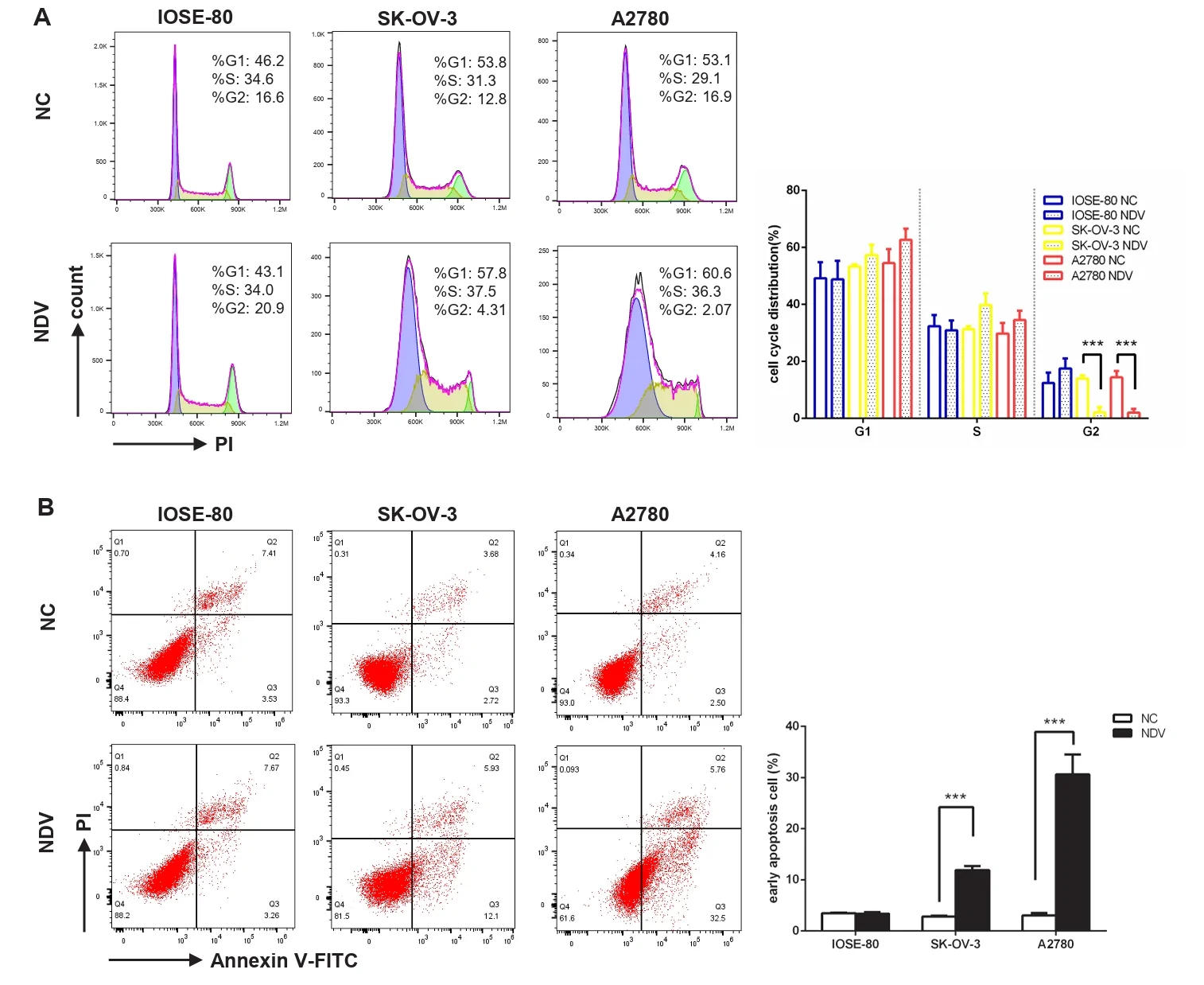
Infection with NDV caused a disrupted cell cycle and prompted apoptosis of ovarian cancer cells. Flow cytometry was performed to explore the influence of NDV on the cell cycle (A) and apoptosis (B) of ovarian cancer cells. The flow cytometry on the left displays a representative experimental result, while the histogram on the right shows the average results of three experiments (^***^, *P* < 0.001; otherwise, *P* > 0.05).

The cell cycle distribution was assessed via flow cytometry. Compared with NC cells, ovarian cancer cells SK-OV-3 and A2780 presented similar cell cycle distributions after infection with NDV: the cell cycle was arrested in G1 and S, with a significant reduction in G2. Infection with NDV reduced the percentage of SK-OV-3 and A2780 cells in the G2 phase from 14.00% to 2.14% and 14.43% to 2.04%, respectively. Interestingly, the percentage of cells in the G2 of IOSE-80 infected with NDV increased from 12.43% to 17.53% (not significantly different) (Fig. 2A).

Apoptosis was measured via Annexin V-FITC/PI flow cytometry analysis. The early apoptosis cell rates of SK-OV-3 and A2780 cells improved after infection with NDV. More severe early apoptosis was induced in A2780 cells, from 3.05% to 30.60%. The early apoptosis cell rate of SK-OV-3 cells was also increased by infection with NDV, from 2.80% to 11.90%. Infection with NDV had no significant influence on IOSE-80 cells (Fig. 2B).

### The migration and invasion of ovarian cancer cells were inhibited by infection with NDV

**Fig. 3.**
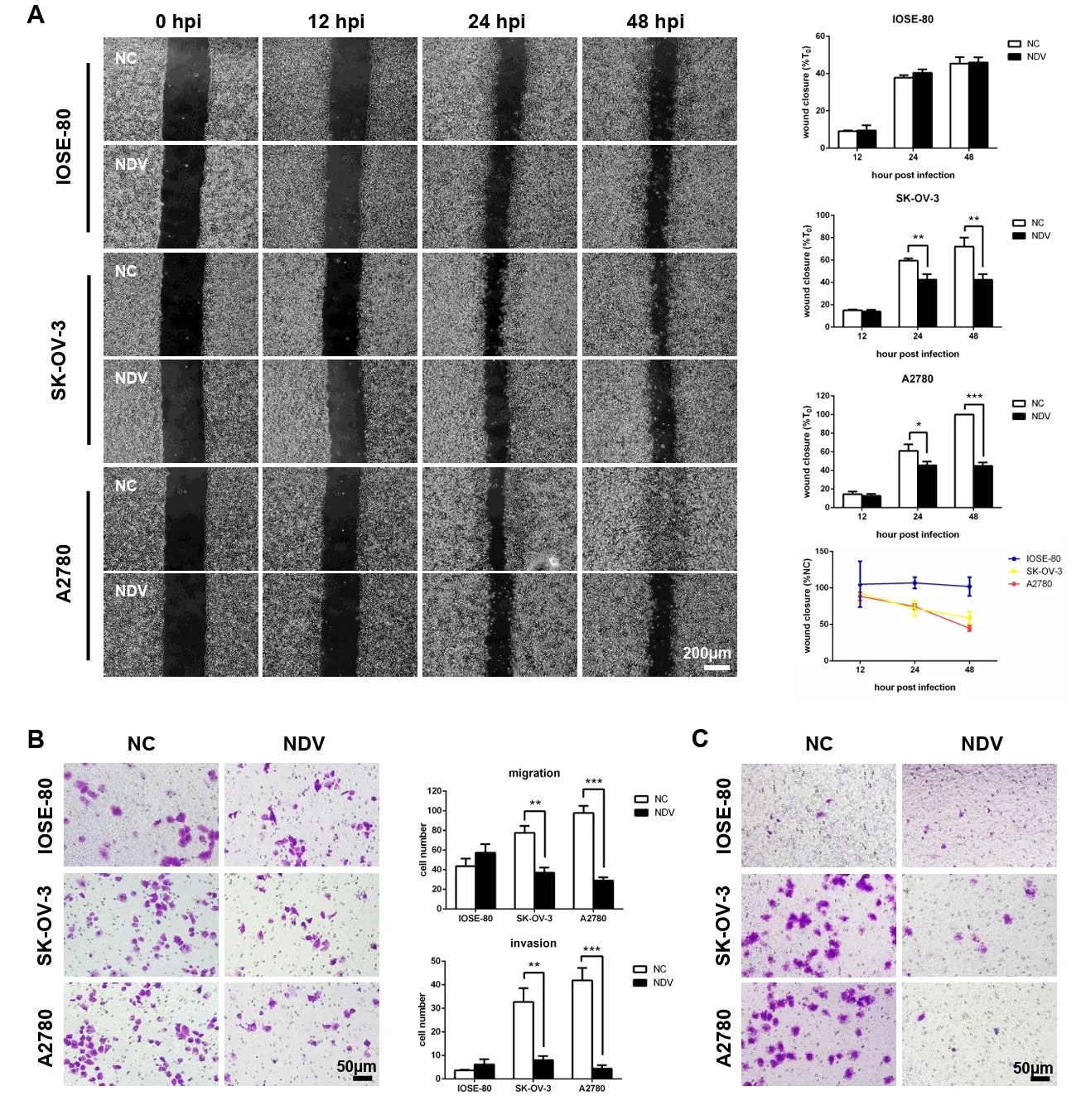
The migration and invasion of ovarian cancer cells were inhibited by infection with NDV. (A) Wound healing assay was carried out to detect the cell migration. Transwell assay was performed. The matrix gel uncoated inserts were used for migration assay (B) and matrix gel coated inserts were used for invasion assay (C) (^*^, *P* < 0.05; ^**^, *P* < 0.01; ^***^, *P* < 0.001; otherwise, *P* > 0.05).

Wound healing and transwell migration assays were used to detect the influence of NDV on ovarian cancer cell migration. Wound healing in NDV-infected SK-OV-3 and A2780 cells was significantly inhibited at 24 and 48 hpi. Compared with that of the NC cells, the percentage of wound closure of the NDV-infected SK-OV-3 and A2780 cells decreased to 71.78% and 74.59% at 24 hpi and 59.10% and 44.87% at 48 hpi, respectively. The values of the NDV-treated IOSE-80 cells were over 100% of those of the NC IOSE-80 cells (Fig. 3A). Transwell migration assays revealed that the migration of both SK-OV-3 and A2780 cells was significantly inhibited by infection with NDV, whereas IOSE-80 cells infected with NDV exhibited more active migration than NC IOSE-80 cells did, but the difference was not significant (Fig. 3B). Transwell invasion assays were performed to determine the effect of NDV on ovarian cancer cell invasion. As shown in Fig. 3C, the number of invaded SK-OV-3 and A2780 cells was also reduced after infection with NDV. These findings suggested that infection with NDV inhibited the migration and invasion of ovarian cancer cells but did not negatively regulate normal ovarian cells.

### DEGs in ovarian cancer cells after infection with NDV and GO/KEGG enrichment analysis

**Fig. 4.**
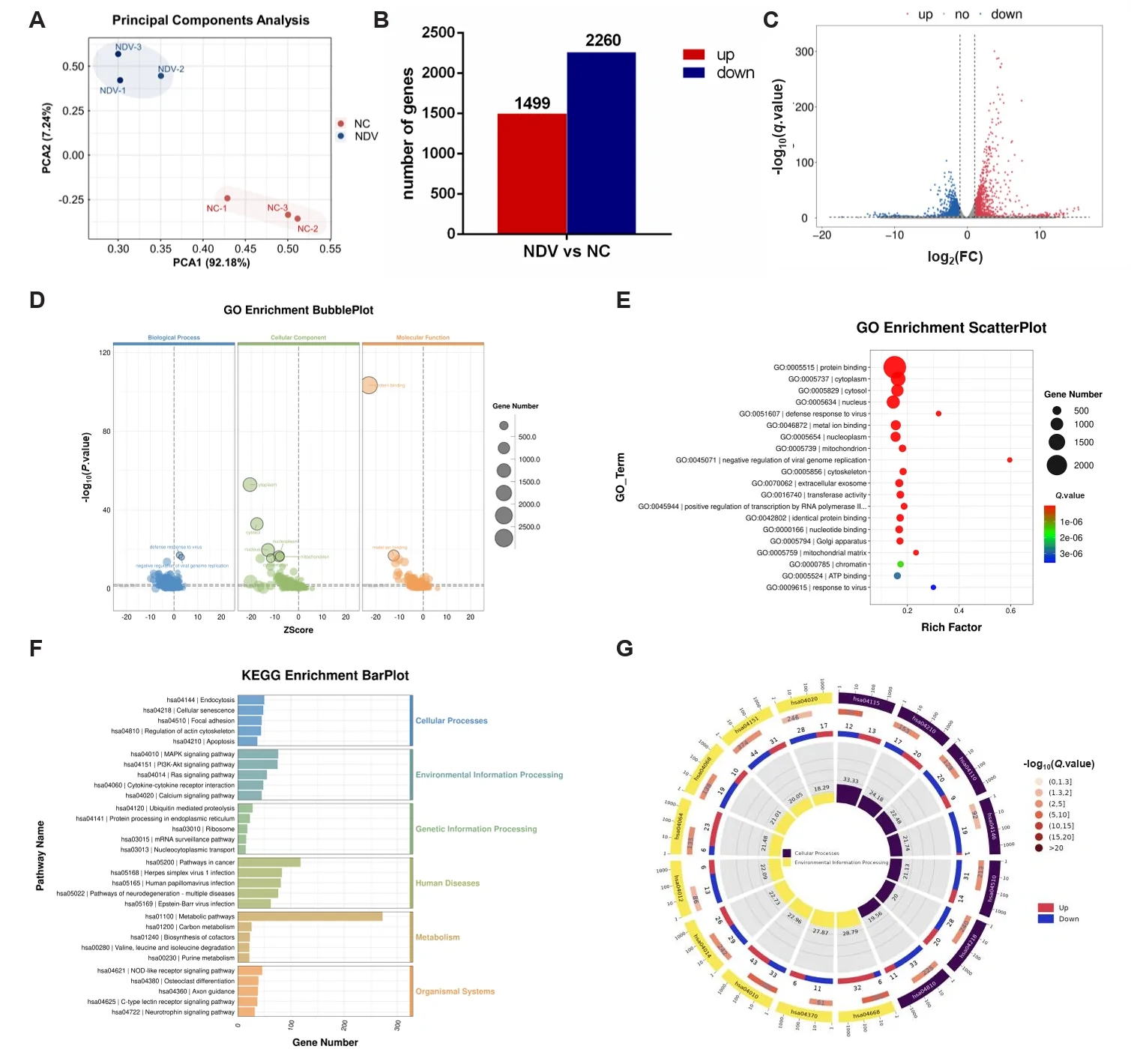
DEGs in ovarian cancer cells A2780 between the NC and NDV-infected groups. (A) Correlation among samples in the NC and NDV groups. (B) Number of significantly up-regulated and down-regulated genes in different groups. (C) Volcano plot of identified genes including up-regulated and down-regulated genes in the RNA-seq. (D) GO enrichment bubbleplot of DEGs. (E) The top 20 enriched significantly differential GO terms of DEGs. (F) KEGG enrichment barplot of DEGs. (G) The significantly enriched KEGG pathways related to the KEGG_Level_1 cellular processes and environment information processing.

To explore the oncolytic mechanism of NDV in ovarian cancer cells, A2780 cells, which are relatively sensitive to NDV, were selected and subjected to transcriptomic sequencing. Principal component analysis (PCA) revealed the high quality of our transcriptome profile data and a distinct separation between the samples from A2780_NDV and A2780_NC (Fig. 4A). Infection with NDV resulted in 3,759 DEGs, including 1,499 upregulated genes and 2,260 downregulated genes (Fig. 4B and 4C).

The functional enrichment of DEGs was carried out to investigate the biological response of ovarian cancer cells to infection with NDV. GO enrichment analysis demonstrated the changes in the categories of biological process (BP), cellular component (CC), and molecular function (MF). In the BP category, the DEGs were associated mainly with the GO terms defense response to virus and negative regulation of viral genome replication. In the CC category, the DEGs were enriched in the GO terms cytoplasm, cytosol, nucleus, nucleoplasm, mitochondrion, and cytoskeleton. In the MF category, the DEGs were related mainly to the GO terms protein binding and metal ion binding (Fig. 4D and 4E).

KEGG enrichment analysis was performed to predict specific pathways that participate in the mechanism of NDV-induced phenotypic changes in ovarian cancer cells. The results showed that 6 KEGG_Level_1 categories were affected (Fig. 4F). On the basis of the results of cell proliferation, cell cycle, apoptosis, migration, and invasion, KEGG_Level_1 cellular processes and environment information processing were screened for further investigation. Among these two Level_1 categories, 13 pathways were significantly enriched (Fig. 4G, Table 1).

### Changes in the pathways related to cell growth and death

**Fig. 5.**
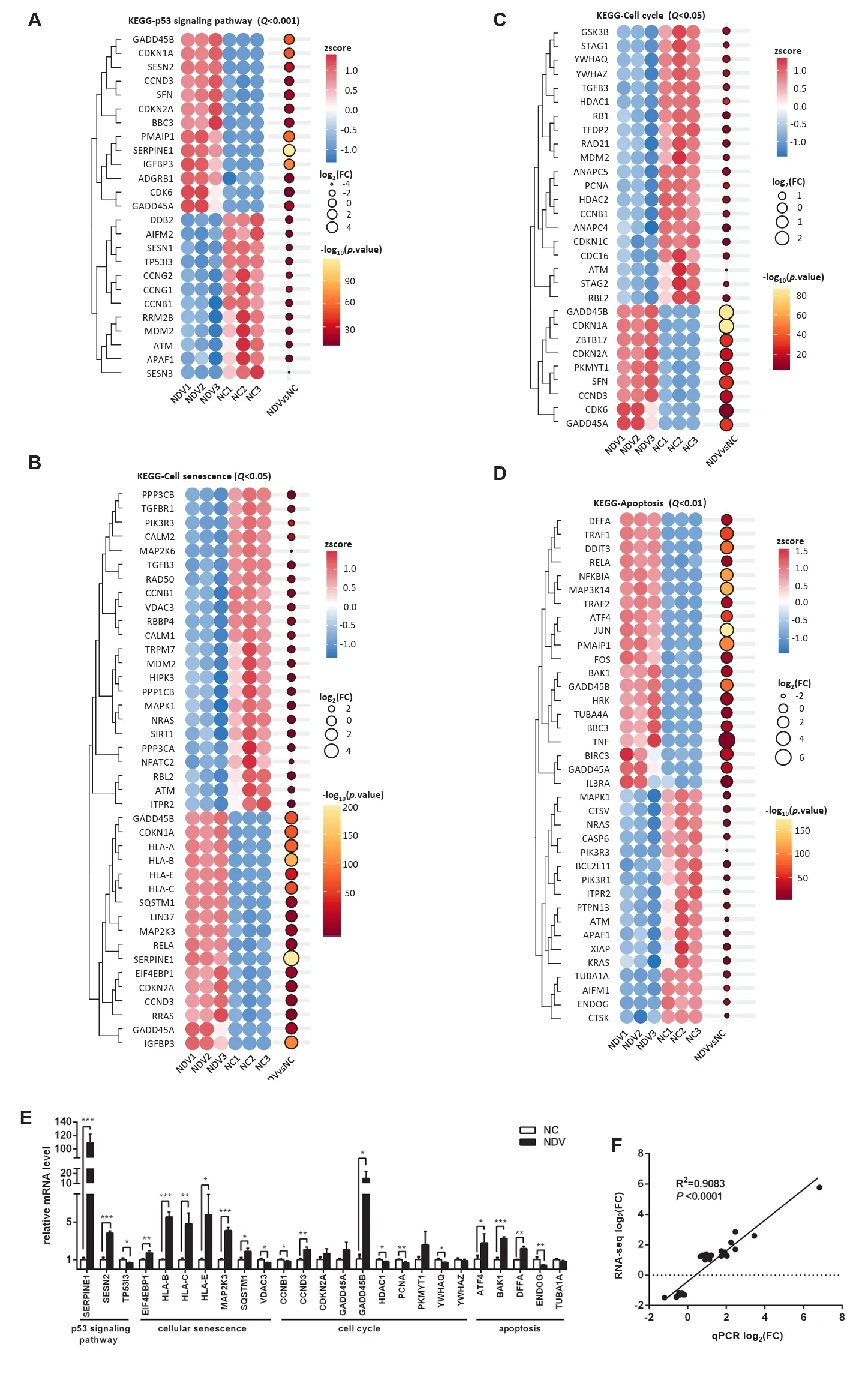
Effect of NDV infection on the pathways related to cell growth and death. DEGs in the pathways p53 signaling pathway (A), cellular senescence (B), cell cycle (C), and apoptosis (D). When the number of DEGs enriched in the pathway is over 40, the top 40 significantly differential DEGs are displayed. (E) The mRNA expression levels of the screened DEGs were detected by qPCR. (F) The correlation between results from qPCR and RNA-seq (^*^, *P* < 0.05; ^**^, *P* < 0.01; ^***^, *P* < 0.001; otherwise, *P* > 0.05).

Infection with NDV inhibited ovarian cancer cell proliferation, disrupted the cell cycle, and promoted apoptosis. The enriched pathways in KEGG_Level_1 cellular processes mainly related to these impacts were the p53 signaling pathway, cellular senescence, cell cycle, and apoptosis, which were significantly different (Table 1). The details of the upregulated and downregulated genes involved in these pathways are shown in Fig. 5A–5D. To further determine the key genes that play important roles in the oncolytic effect of NDV on ovarian cancer cells, genes with FPKM ≥10 in both the experimental and control groups were screened (Table S2) and subjected to qPCR to validate the reliability of the transcriptome sequencing results. The mRNA levels of 25 genes involved in the 4 pathways were subsequently detected (Fig. 5E). Correlation analysis revealed that the qPCR data were significantly consistent with the RNA-seq data (R^2^ =0.9083, *P* < 0.0001; Fig. 5F).

### Changes in the pathways related to cell migration and invasion

**Fig. 6.**
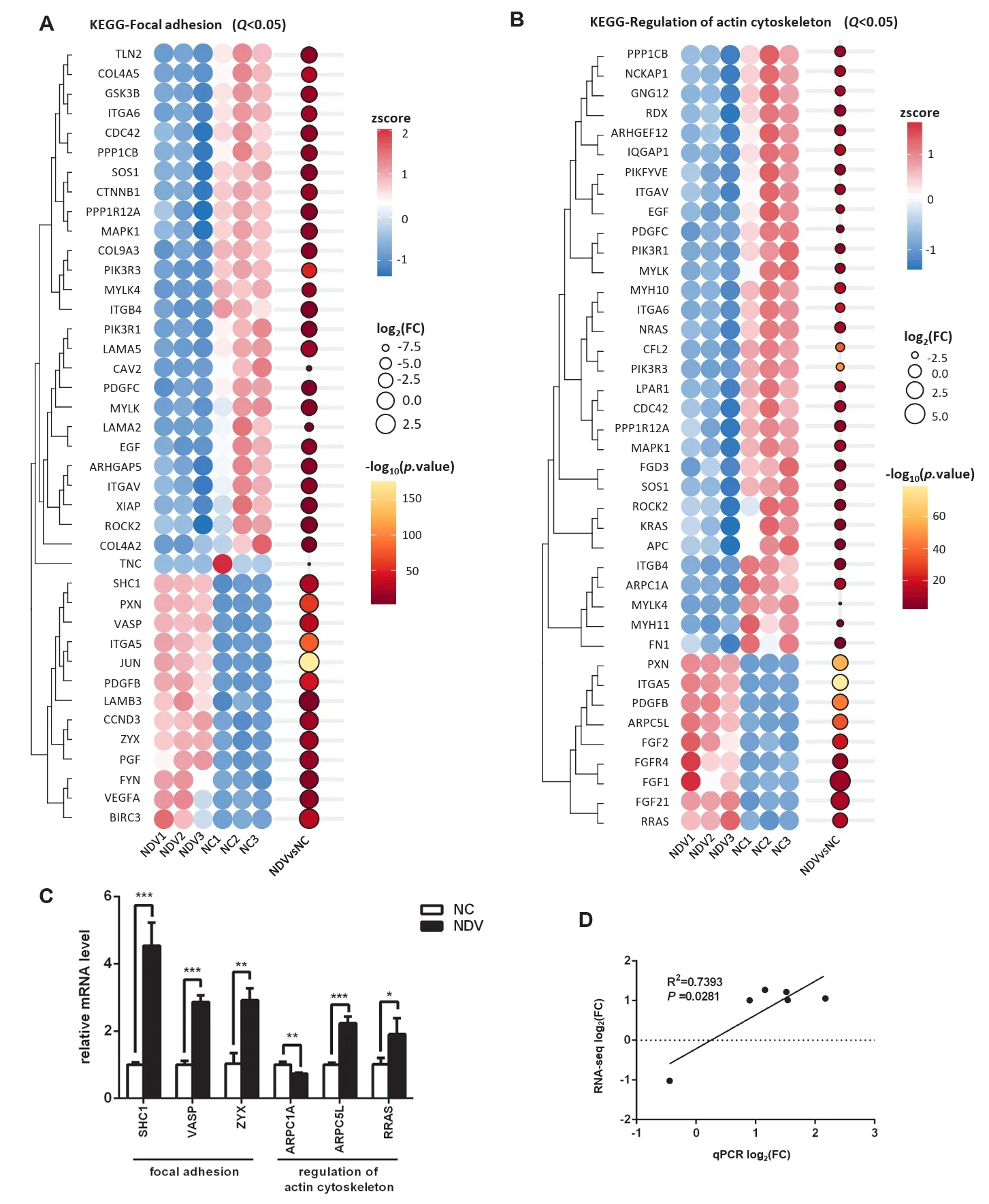
Effect of NDV infection on the pathways related to cell migration and invasion. DEGs in the pathways focal adhesion (A) and regulation of actin cytoskeleton (B). When the number of DEGs enriched in the pathway is over 40, the top 40 significantly differential DEGs are displayed. (C) The mRNA expression levels of the screened DEGs were detected by qPCR. (D) The correlation between results from qPCR and RNA-seq (^*^, *P* < 0.05; ^**^, *P* < 0.01; ^***^, *P* < 0.001; otherwise, *P* > 0.05).

Infection with NDV caused the pathways focal adhesion and regulation of actin cytoskeleton in A2780 cells to be significantly different from those in NC cells (Table 1). Detailed information on the upregulated and downregulated genes related to these pathways is shown in Fig. 6A and 6B. To further determine the key genes involved in the influence of NDV on ovarian cancer cell migration and invasion, 6 genes with FPKM ≥10 in both the experimental and control groups were screened via qPCR (Fig. 6C, Table S2). Further correlation analysis revealed a significant positive relationship between the qPCR and RNA-seq results (R^2^ =0.7393, *P* = 0.0281; Fig. 6D).

### Changes in the pathways related to signal transduction

**Fig. 7.**
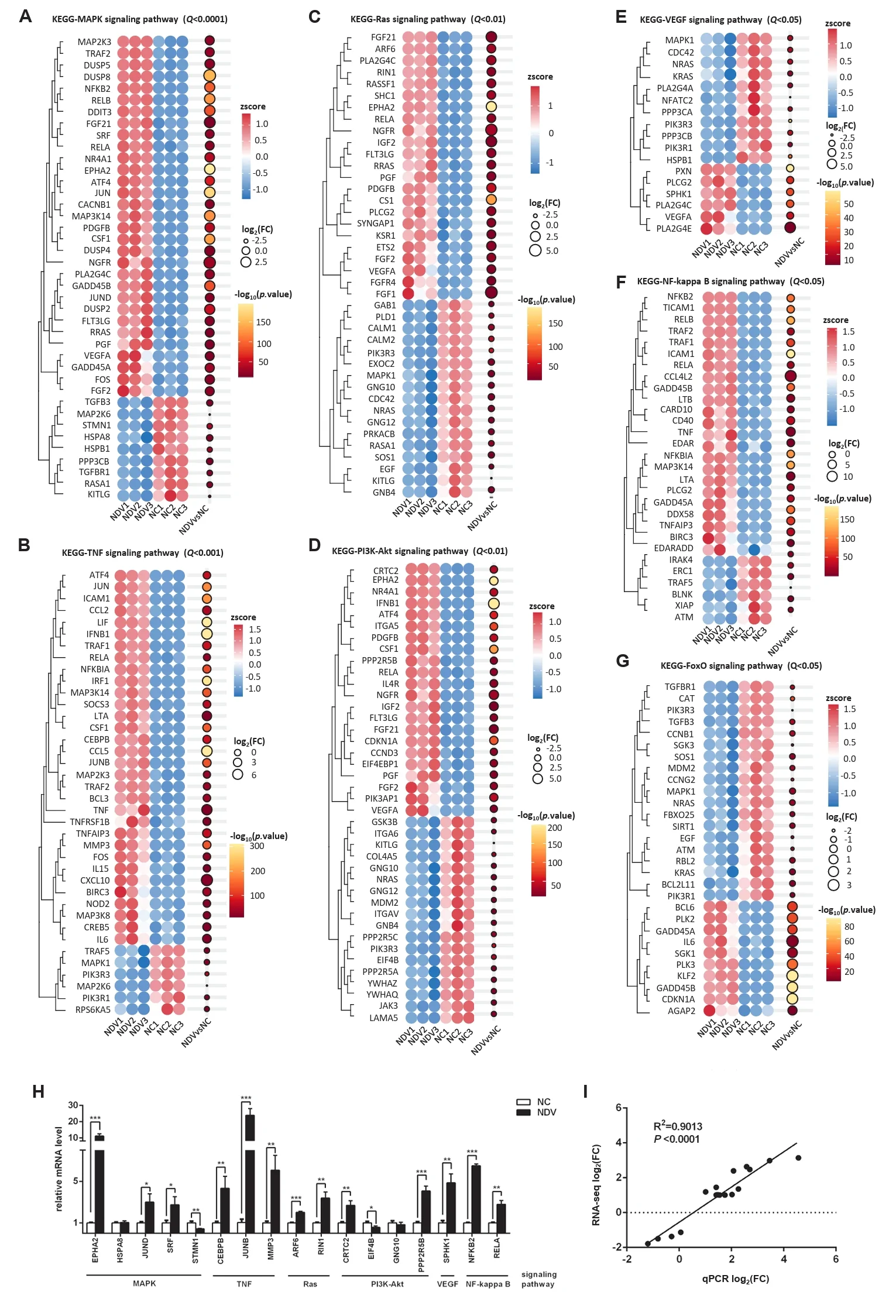
Effect of NDV infection on the pathways related to signal transduction. DEGs in the pathways MAPK signaling pathway (A), TNF signaling pathway (B), Ras signaling pathway (C), PI3K-Akt signaling pathway (D), VEGF signaling pathway (E), NF-kappa B signaling pathway (F), and FoxO signaling pathway (G). When the number of DEGs enriched in the pathway is over 40, the top 40 significantly differential DEGs are displayed. (H) The mRNA expression levels of the screened DEGs were detected by qPCR. (I) The correlation between results from qPCR and RNA-seq (^*^, *P* < 0.05; ^**^, *P* < 0.01; ^***^, *P* < 0.001; otherwise, *P* > 0.05).

Tumor progression and regression are dependent on complicated signal transduction. Among the enriched pathways associated with KEGG_Level_1 Environmental Information Processing, the MAPK signaling pathway, TNF signaling pathway, Ras signaling pathway, PI3K-Akt signaling pathway, VEGF signaling pathway, NF-kappa B signaling pathway, and FoxO signaling pathway, which were significantly different, were screened (Table 1). The details of the upregulated and downregulated genes related to these pathways are shown in Fig. 7A–7G. Next, 17 genes were selected on the basis of FPKM ≥10 in both the experimental and control groups (Table S2), and the mRNA levels of 17 genes were detected via qPCR (Fig. 7H). Correlation analysis revealed a significantly positive relationship between the qPCR and RNA-seq results (R^2^ =0.9013, *P* < 0.0001; Fig. 7I).

### The gene-pathway and PPI networks

**Fig. 8.**
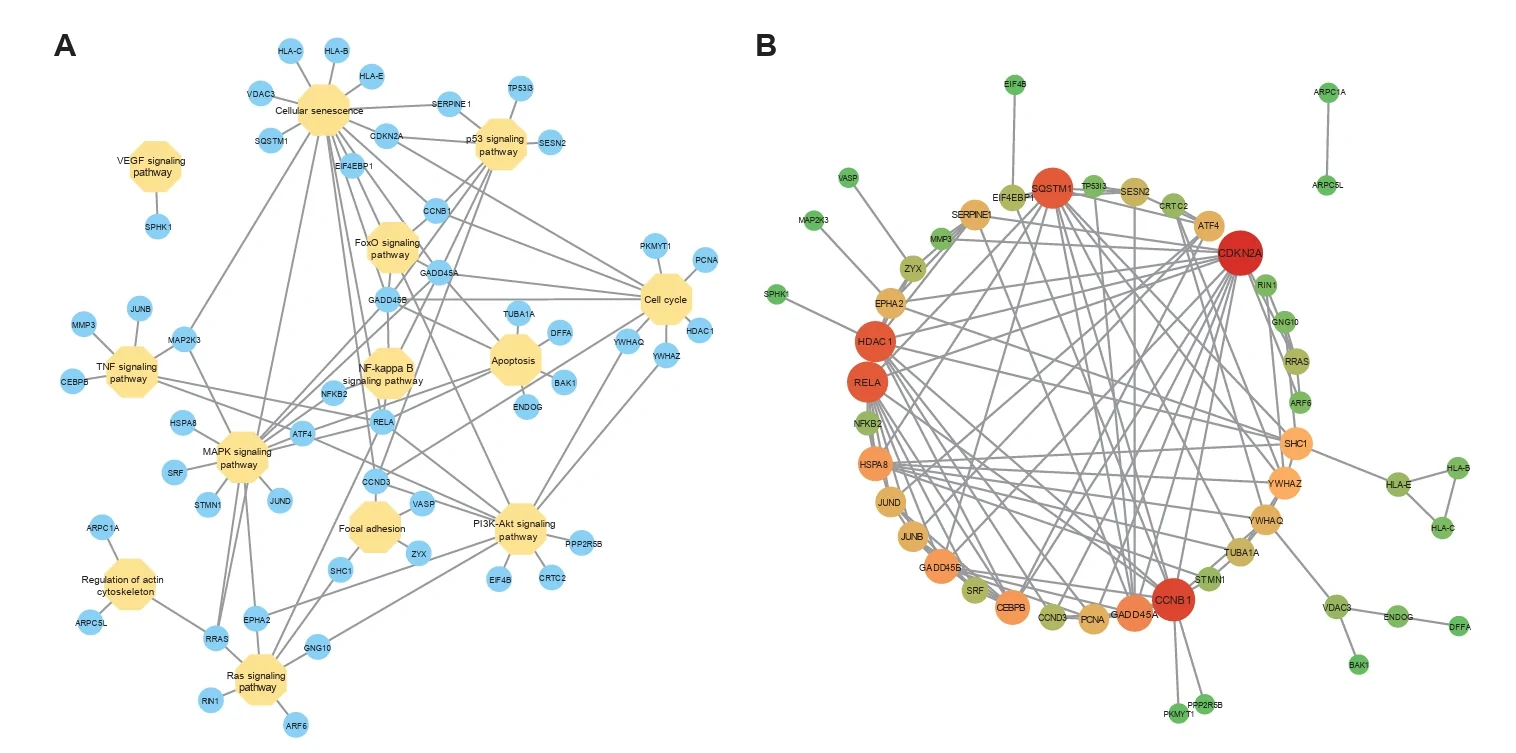
The interaction among pathways and DEGs. (A) The gene-pathway network showed the connection among pathways via some DEGs. (B) The PPI network indicated the interaction among DEGs, helping us find the relatively important DEGs.

Although we artificially classified the 48 desired DEGs into the pathways mentioned above, some DEGs were active in multiple pathways and interacted with each other. The gene-pathway network indicated that some genes were related to more than one pathway. For example, the genes GADD45A, GADD45B, and RELA were related to 7 pathways (Fig. 8A). The PPI network helped us identify more important genes involved in the oncolytic effect of NDV in ovarian cancer cells. The results revealed that the genes CDKN2A, CCNB1, SQSTM1, HDAC1, and RELA participated in relatively more interactions among the 13 screened KEGG pathways involved in NDV-induced phenotypic changes in ovarian cancer cells, which calls for investigating the specific functions of these genes in the future (Fig. 8B).

### The expression level of GADD45B in cells A2780 and IOSE-80

**Fig. 9.**
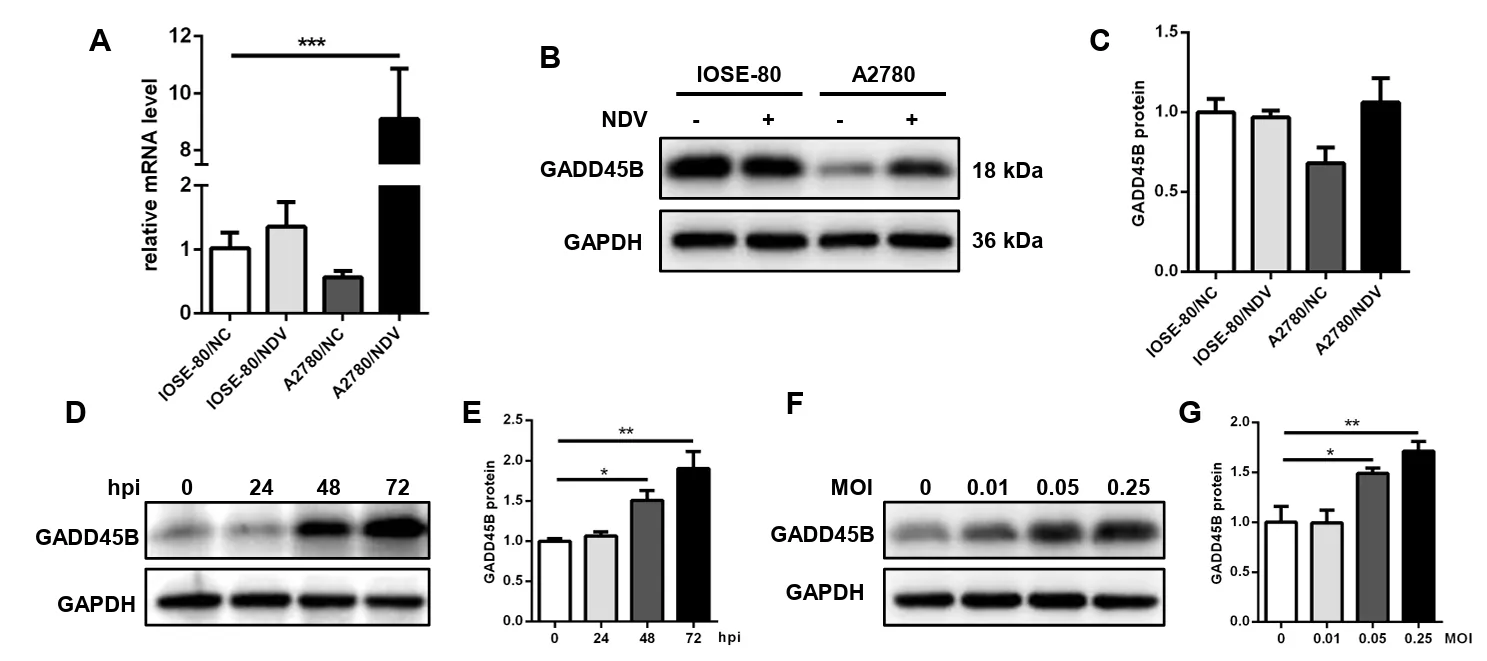
The expression level of GADD45B in cells A2780 and IOSE-80. The mRNA expression level (A) and protein expression level (B, C) of GADD45B in A2780 and IOSE-80. The protein expression of GADD45B in A2780 showed a positive correlation with hpi (D, E) and MOI of NDV infection (F, G) (^*^, *P* < 0.05; ^**^, *P* < 0.01; ^***^, *P* < 0.001; otherwise, *P* > 0.05).

According to the results of qPCR, the expression of GADD45B is obviously enhanced in cells A2780 by infection of NDV (Fig. 5). The gene-pathway and PPI networks showed that GADD45B was involved in the most pathways and interacted with more other DEGs (Fig. 8). qPCR and Western Blot were employed to confirm whether the expression change was induced in IOSE-80 by infection with NDV. The results suggested that the expression level of GADD45B in A2780 is lower than that in IOSE-80 and the expression level of GADD45B in A2780 increases with the hpi and MOI of NDV infection (Fig. 9).

## Discussion

Ovarian cancer, the eighth most common cancer in women and the second leading cause of gynecologic cancer mortality worldwide, poses the challenge of serious chemoresistance, presenting a significant hurdle (Webb and Jordan, 2024). NDV can selectively kill neoplastic cells while replicating without harming normal cells (Reichard et al., 1992). Although the use and mechanism of NDV virotherapy in the treatment of ovarian cancer have not been extensively investigated, the few studies available have shown definite potential (Ahlert et al., 1997; Matuszewska et al., 2019; Rangaswamy et al., 2017). In this study, the human ovarian cancer cell lines SK-OV-3 and A2780 were infected with NDV to investigate the oncolytic activity of NDV, and the normal ovarian cell line IOSE-80 was used as a control. Phenotypes such as cell proliferation, cell cycle, apoptosis, migration, and invasion were detected. We further performed transcriptomic analysis to determine the key pathways and genes that contribute significantly to the phenotypic changes in NDV-treated cells.

The replication ability of NDV in ovarian cancer cells is up to 1000 times greater than that in normal ovarian cells, which verifies the selectivity of NDV (Fig. 1). This is likely because tumor cells have defects in the activation of antiviral signaling pathways (Schirrmacher, 2022), type I IFN signaling pathways (Gal-Ben-Ari et al., 2018), apoptotic pathways, Ras signaling, and the expression of the Rac1 protein (Schirrmacher, 2015). Moreover, human tumor cells infected with NDV exhibit high-density cell surface expression of the viral HN and F proteins, promoting NDV binding and entry into tumor cells (Washburn and Schirrmacher, 2002). By detecting the cell proliferation, cell cycle, apoptosis, migration, and invasion of human ovarian cancer cells after infection with NDV, we confirmed that the proliferation, migration, and invasion of both ovarian cancer cells SK-OV-3 and A2780 were inhibited; the cell cycle was disrupted; and apoptosis was improved. Interestingly, enhanced proliferation, migration, and invasion were detected when normal ovarian cells IOSE-80 were infected with NDV. Additionally, more IOSE-80 cells entered the G2 phase, which may have contributed to improved proliferation, migration, and invasion (Figs. 2 and 3). Although the results did not significantly differ from those of the NC cells, this phenomenon provided evidence that NDV was harmless to normal cells and even positive to them, which is good news for utilizing NDV virotherapy in the treatment of ovarian cancer.

In addition to the classic obstacles caused by p53 in cancer development, including cell cycle arrest, apoptosis, and senescence, an increasing number of new functions of the p53 signaling pathway have been discovered, such as metabolism, ferroptosis, immunity, and others, that contribute to tumor suppression (Liu et al., 2024). Notably, both the SERPINE1 and SESN2 genes, which are involved in the p53 signaling pathway, were dramatically upregulated in NDV-infected ovarian cancer cells, especially gene SERPINE1, with an mRNA expression level up to 109.11 times greater than that in NC cells, and with the maximum | log_2_(FC) | value among all DEGs. SERPINE1 has been proven to promote tumor progression in various cancers and facilitate tumor cell proliferation, migration, and invasion (Li et al., 2023; Yang et al., 2019). SERPINE1 knockdown in cisplatin-resistant derivative A2780cp cells inhibited the epithelial‑mesenchymal transition (EMT) process (Pan et al., 2017). However, in this study, the expression of SERPINE1 was upregulated, which contrasts with the observed inhibition of ovarian cancer progression. A previous study revealed a direct antiviral function of the gene SERPINE1 (Dittmann et al., 2015), which suggested that the extremely high expression of SERPINE1 may be due to the antiviral response of ovarian cancer cells to NDV. SESN2 has been reported to suppress tumorigenesis in a variety of cancers. SESN2 suppressed the proliferation of colorectal cancer cells by activating the AMPK pathway and inhibiting mTORC1 signaling (Wei et al., 2017). Conversely, SESN2 knockdown can promote endometrial cancer cell proliferation, migration, and ROS production, which has also been confirmed in a xenograft nude mouse model (Shin et al., 2020). In our study, the significant increase in SESN2 induced by NDV may mediate the oncolytic effect of NDV on ovarian cancer cells SK-OV-3 and A2780 via the inhibition of proliferation and migration.

Infection with NDV caused the expression of the human leukocyte antigens (HLAs) B/C/E, which belong to the HLA (also named human major histocompatibility complex) class Ⅰ, are markedly upregulated in ovarian cancer cells A2780 (Fig. 5). According to the KEGG annotation, HLA class Ⅰ could facilitate DNA damage. Moreover, research has shown that DNA damage enhances the presentation of HLA class I on the surface of damaged cells (Uchihara and Shibata, 2023). HLA class Ⅰ and DNA damage may be mutually regulated. Moreover, as an independent factor in the presentation of tumor-associated antigens, HLA class Ⅰ plays an important role in the antitumor immune response. HLA class Ⅰ-negative status helps tumor cells escape immune systems. More diverse HLA class I genes lead to a wider range of antigen presentation, increasing the likelihood of presenting more immunogenic antigens and increasing the likelihood of benefiting from immune checkpoint inhibitors (ICIs). Modulating DNA damage-induced HLA class I antigen presentation may regulate immune cell infiltration and immune checkpoint proteins (Okami et al., 2023). Cellular senescence is considered to be immunogenic and related to HLA class I (Marin et al., 2023). Hence, NDV-induced upregulation of HLA B/C/E in ovarian cancer cells likely participates in direct damage and immune regulation.

The activation status of cell cycle pathways is a key factor affecting tumor growth. Infection with NDV resulted in a lower proportion of ovarian cancer cells in the G2 phase. The qPCR results revealed that the mRNA level of GADD45B was approximately 15 times greater than that of the negative control (Fig. 5). When GADD45B is overexpressed in melanoma cells, the number of cells in the G2 phase significantly decreases (Chen et al., 2023). According to the PPI network, CDKN2A may be a key protein involved in the oncolytic effect of NDV (Fig. 8). CDKN2A is confirmed to be directly involved in TP53-dependent cell cycle regulation pathways, and it is likely upstream of the signal axis, affecting the expression of TP53 and GADD45B (Chen et al., 2023; Deneka et al., 2022). In addition, the overexpression of GADD45B can inhibit the proliferation, migration, and invasion and increase the apoptosis of gastric cancer cells (Li et al., 2023). GADD45B has been found to have lower expression levels in ovarian cancer tissues compared to normal tissues (Zhan et al., 2024), which is consistent with our research in ovarian cancer cells and normal ovarian epithelial cells (Fig. 9). In addition, the expression increase of GADD45B in cells A2780 was positively correlated with hpi and MOI of NDV infection, which implied the important role of GADD45B in the NDV’s oncolytic effect on ovarian cancer and should be confirmed in the future through functional assays.

ATF4, BAK1, and DFFA, which are involved in the KEGG apoptosis pathway, were upregulated in NDV-infected A2780 cells (Fig. 5). ATF4 is a member of the ATF/CREB family and plays a critical role as a stress-induced transcription factor. Apoptosis can be triggered by ATF4 via many axes, such as the mTOR-EIF2AK3-ATF4-DDIT3 axis and the PERK-ATF4-CHOP axis (Kim and Ko, 2021; Tang et al., 2024). NDV primarily triggers the activation of the intrinsic mitochondrial apoptosis pathway, leading to programmed cell death (Elankumaran et al., 2006). BAK1 is a critical regulator of mitochondrial apoptosis, and the upregulation of BAK1 induces apoptosis (Yang et al., 2018). As a major apoptotic endonuclease for DNA fragmentation, DFFA triggers DNA fragmentation during apoptosis (Zhang and Xu, 2000). Thus, the NDV-induced upregulation of ATF4, BAK1, and DFFA may promote the apoptosis of ovarian cancer cells.

EMT is an important stage in tumor development. After undergoing EMT, tumor cells acquire migratory and invasive features and infiltrate the surrounding matrix, forming a microenvironment that promotes tumor growth and metastasis. Infection with NDV inhibited the migration and invasion of ovarian cancer cells (Fig. 3). In the focal adhesion pathway, the gene ZYX, which can inhibit proliferation, migration, invasion, and EMT in gastric cancer cells, was upregulated, suggesting that ZYX may be related to the oncolytic process of NDV in ovarian cancer cells (Lou et al., 2020).

Multiple signal transduction modes and pathways are known to exist within cells, and multiple levels of cross-regulation exist between these modes and pathways, synthesizing a highly complex network system. The gene-pathway network revealed the connections among pathways by genes (Fig. 8A). In this study, the MAPK signaling pathway, TNF signaling pathway, Ras signaling pathway, PI3K-Akt signaling pathway, VEGF signaling pathway, NF-kappa B signaling pathway, and FoxO signaling pathway in ovarian cancer cells were significantly regulated by infection with NDV (Table 1). These pathways interact with each other during the NDV-induced inhibition of cell proliferation, migration, and invasion; cell cycle arrest; and promotion of apoptosis. For example, the gene RELA, which is outstanding in the PPI network, is enriched not only in the well-known classic NF-kappa B signaling pathway but also in the TNF signaling pathway, MAPK signaling pathway, Ras signaling pathway, and PI3K-Akt signaling pathway. However, there is controversy over the functionality of RELA. On the one hand, RELA limits TRAIL-induced apoptosis in pancreatic cancer (Geismann et al., 2023). On the other hand, in colon, breast, and prostate cancer cells, RELA functions as a tumor suppressor by inducing apoptosis and senescence (Bu et al., 2016). Therefore, the role of RELA in ovarian cancer and its role in the oncolytic effect of NDV are worth studying.

The TNF signaling pathway regulates key cellular processes such as apoptosis and proliferation in addition to its well-known role in the immune response through the activation of various intracellular signaling pathways (such as the MAPK, Akt, and NF-kappa B pathways), indicating the multiple roles of these pathways in the mechanism underlying the oncolytic activity of NDV in ovarian cancer cells (Manohar, 2024). Because NDV can not only directly kill cancer cells but also activate both innate and adaptive immunity and regulate the tumor TME.

In this study, ovarian cancer cells were infected with NDV, and the results verified the selective propagation and killing of NDV. Transcriptomic sequencing was carried out to explore the mechanism of NDV-induced phenotypic changes in ovarian cancer cells. Through transcriptomic analysis, several pathways and genes have attracted our interest. This study briefly discussed several enriched KEGG pathways and pointed out certain genes, especially SESN2, HLA B/C/E, GADD45B, and RELA, that may play crucial roles in the oncolytic effect of NDV on ovarian cancer cells. The function of the upregulated or downregulated enriched genes should be determined by a series of functional experiments in the future to determine the specific mechanisms by which infection with NDV suppresses the development of ovarian cancer. This study presents a direction for future research and provides an effective basis for screening genes that effectively participate in the oncolytic effect of NDV on ovarian cancer cells and for the subsequent design of small molecule inhibitors or activators during virotherapy of ovarian cancer. In addition, the enriched genes related to the immune response could be potential targets for combining virotherapy and immunotherapy for ovarian cancer to improve the immunotherapy response rate and prolong the survival time of ovarian cancer patients.

## Figures and Tables

**Table 1.** Thirteen important pathways involved in NDV-induced phenotypical changes in ovarian cancer cells

Pathway_ID	KEGG_Level_2	Pathway_Name	Rich. Factor	S_Up_Num	S_Down_Num	*P*.value	*Q*.value
hsa04010	Signal transduction	MAPK signaling pathway	0.2296	43	33	1.21967E-06	8.0742E-05
hsa04668	Signal transduction	TNF signaling pathway	0.2879	32	6	2.6516E-06	0.000146280
hsa04115	Cell growth and death	p53 signaling pathway	0.3333	13	12	8.36252E-06	0.000395428
hsa04014	Signal transduction	Ras signaling pathway	0.2273	26	29	4.88055E-05	0.001615463
hsa04210	Cell growth and death	Apoptosis	0.2418	20	17	0.000222989	0.003354967
hsa04151	Signal transduction	PI3K-Akt signaling pathway	0.2005	31	44	0.000198133	0.003354967
hsa04510	Cellular community - eukaryotes	Focal adhesion	0.2113	14	31	0.001213301	0.013386752
hsa04370	Signal transduction	VEGF signaling pathway	0.2787	6	11	0.002229870	0.019138009
hsa04218	Cell growth and death	Cellular senescence	0.2000	20	28	0.002810453	0.022994128
hsa04110	Cell growth and death	Cell cycle	0.2248	9	20	0.003335481	0.024048231
hsa04810	Cell motility	Regulation of actin cytoskeleton	0.1956	11	33	0.006280651	0.037798102
hsa04064	Signal transduction	NF-kappa B signaling pathway	0.2148	23	6	0.006583374	0.038912446
hsa04068	Signal transduction	FoxO signaling pathway	0.2101	10	19	0.009004390	0.048269160

## Supplementary Information

Table S1. Primer sequences in qPCR

Table S2. The expression level of screened 48 DEGs
