# Supplementary material for: The key pathways and genes related to oncolytic Newcastle disease virus-induced phenotypic changes in ovarian cancer cells
Source: J Microbiol. 2025 Apr 29;63(4):e2411018. doi: 10.71150/jm.2411018 (PMC13577072; doi:10.71150/jm.2411018)
Supplement: Supplementary file 1 [file jm-2411018-Supplementary-Table-S1.pdf]

Table S1. Primer sequences in qPCR

Name	Forward primer Sequence (5′-3′)	Reverse primer Sequence (5′-3′)
GAPDH	GTCTCCTCTGACTTCAACAGCG	ACCACCCTGTTGCTGTAGCCAA
ARF6	CCAAGGTCTCATCTTCGTAGTGG	AGGTCCTGCTTGTTGGCGAAGA
ARPC1A	AGAGTTCCTGCCGCTCCTAAGT	ACTTGGAGACGAAGGTCAGGCA
ARPC5L	TTGCGGAACTCTCCCGTCAACA	CAAGTCAACGCCGTTTCTGTCC
ATF4	TTCTCCAGCGACAAGGCTAAGG	CTCCAACATCCAATCTGTCCCG
BAK1	TTACCGCCATCAGCAGGAACAG	GGAACTCTGAGTCATAGCGTCG
CCNB1	GACCTGTGTCAGGCTTTCTCTG	GGTATTTTGGTCTGACTGCTTGC
CCND3	AGATCAAGCCGCACATGCGGAA	ACGCAAGACAGGTAGCGATCCA
CDKN2A	CTCGTGCTGATGCTACTGAGGA	GGTCGGCGCAGTTGGGCTCC
CEBPB	AGAAGACCGTGGACAAGCACAG	CTCCAGGACCTTGTGCTGCGT
CRTC2	GGCAGTCTCATTATGGGACACC	TGATGGGCTCTCCATGCTGAAC
DFFA	GAGAGGAAGTGCGTCAGTCCAA	GTGTCTACTGCATCCACCTCCT
EIF4B	GGCAAAGCCTGTTGACACAGCT	TTCACTTCGCCAGCTTGGGTGT
EIF4EBP1	CAAGGGATCTGCCCACCATT	AACTGTGACTCTTCACCGCC
ENDOG	CACCTCAACCAGAATGCCTGGA	GTAGGATTTCCCATCAGCCTCTG
EPHA2	ACTGCCAGTGTCAGCATCAACC	GTGACCTCGTACTTCCACACTC
GADD45A	CTGGAGGAAGTGCTCAGCAAAG	AGAGCCACATCTCTGTCGTCGT
GADD45B	GCCAGGATCGCCTCACAGTGG	GGATTTGCAGGGCGATGTCATC
GNG10	GCGTGGAGAGGATCAAGGTCT	CGGAAGGGGTTACTTCCAGC
HDAC1	GGTCCAAATGCAGGCGATTCCT	TCGGAGAACTCTTCCTCACAGG
HLA-B	CTGCTGTGATGTGTAGGAGGAAG	GCTGTGAGAGACACATCAGAGC
HLA-C	GGAGACACAGAAGTACAAGCGC	ACATCCTCTGGAGGGTGTGAGA
HLA-E	CGGCTACTACAATCAGAGCGAG	AATCCTTGCCGTCGTAGGCGAA
HSPA8	TCCTACCAAGCAGACACAGACC	CAGGAGGTATGCCTGTGAGTTC
JUNB	CGATCTGCACAAGATGAACCACG	CTGCTGAGGTTGGTGTAAACGG
JUND	ATCGACATGGACACGCAGGAGC	CTCCGTGTTCTGACTCTTGAGG
MAP2K3	CTTGGTGACCATCTCAGAACTGG	CTTCTGCTCCTGTGAGTTCACG
MMP3	CACTCACAGACCTGACTCGGTT	AAGCAGGATCACAGTTGGCTGG
NFKB2	GGCAGACCAGTGTCATTGAGCA	CAGCAGAAAGCTCACCACACTC
PCNA	CAAGTAATGTCGATAAAGAGGAGG	GTGTCACCGTTGAAGAGAGTGG
PKMYT1	TATGGGACAGCAGCGGATGTGT	AGAACGCAGCTCGGAAGACAGA
PPP2R5B	CTGAAGACGGAGCACAAGCAGT	TCAGAGTGGCATCCTTCTCCAG
RELA	TGAACCGAAACTCTGGCAGCTG	CATCAGCTTGCGAAAAGGAGCC
RIN1	ATGCGGTTGCCTGAAGCCAGTG	CAGTAGGCACAGATGAGCTGGA
RRAS	CTGCTGGTGTTCGCCATTAACG	GATCTGCCTTGTTCCCGACCAA
SERPINE1	CTCATCAGCCACTGGAAAGGCA	GACTCGTGAAGTCAGCCTGAAAC
SESN2	AGATGGAGAGCCGCTTTGAGCT	CCGAGTGAAGTCCTCATATCCG
SHC1	ACAGCCGAGTATGTCGCCTATG	CAATGGTGCTGATGACATCCTGG
SPHK1	GCTGGCAGCTTCCTTGAACCAT	GTGTGCAGAGACAGCAGGTTCA
SQSTM1	TGTGTAGCGTCTGCGAGGGAAA	AGTGTCCGTGTTTCACCTTCCG
SRF	TCACCTACCAGGTGTCGGAGTC	GTGCTGTTTGGATGGTGGAGGT

Name	Forward primer Sequence (5′-3′)	Reverse primer Sequence (5′-3′)
STMN1	AGAACCGAGAGGCACAAATGGC	TCTCGTCAGCAGGGTCTTTGGA
TP53I3	CACCAGTTTGCTGAGGTCTAGG	CCTGGATTTCGGTCACTGGGTA
TUBA1A	CGGGCAGTGTTTGTAGACTTGG	CTCCTTGCCAATGGTGTAGTGC
VASP	CTGGGAGAAGAACAGCACAACC	AGGTCCGAGTAATCACTGGAGC
VDAC3	CCATAAACCTTGCTTGGACAGCT	CCAGTCCAATCAGGCTGGCATT
YWHAQ	AAGTGGAGTCCGAGCTGAGATC	CACACGCAACTTCAGCAAGGTAC
YWHAZ	ACCGTTACTTGGCTGAGGTTGC	CCCAGTCTGATAGGATGTGTTGG
ZYX	TTCCACATCGCCTGCTTCACCT	CGCAGGTGTTACACTTCTCCAG
