# Supplementary material for: The key pathways and genes related to oncolytic Newcastle disease virus-induced phenotypic changes in ovarian cancer cells
Source: J Microbiol. 2025 Apr 29;63(4):e2411018. doi: 10.71150/jm.2411018 (PMC13577072; doi:10.71150/jm.2411018)
Supplement: Supplementary file 2 [file jm-2411018-Supplementary-Table-S2.pdf]

Table S2. The expression level of screened 48 DEGs

Gene name	FPKM. NDV_1	FPKM. NDV_2	FPKM. NDV_3	FPKM. NC_1	FPKM. NC_2	FPKM. NC_3	log_2(FC) by RNA-seq	log_2(FC) by qPCR
ARF6	30.65	30.93	31.16	13.18	14.37	13.05	1.19	1.01
ARPC1A	31.39	31.16	25.98	65.42	60.19	54.10	-1.02	-0.45
ARPC5L	38.05	34.80	31.89	15.48	14.07	13.83	1.27	1.16
ATF4	229.61	219.04	200.00	74.53	73.61	68.08	1.58	1.75
BAK1	63.42	65.95	73.34	28.36	27.52	28.86	1.26	1.75
CCNB1	22.66	21.15	12.96	48.27	48.38	43.07	-1.30	-0.20
CCND3	23.86	24.67	26.59	12.55	10.39	11.55	1.12	1.07
CDKN2A	26.65	25.97	29.92	12.11	11.29	11.95	1.22	0.66
CEBPB	71.08	69.53	78.08	15.01	12.55	14.06	2.39	2.08
CRTC2	27.03	27.12	25.19	10.39	9.20	9.45	1.45	1.42
DFFA	23.18	22.84	22.28	10.96	11.35	10.75	1.05	1.13
EIF4B	24.38	24.98	17.18	58.55	65.03	64.17	-1.50	-0.81
EIF4EBP1	319.09	306.45	360.70	151.37	118.24	132.19	1.30	0.78
ENDOG	48.59	49.27	50.52	149.26	128.95	136.23	-1.48	-1.20
EPHA2	90.68	92.14	82.97	11.17	11.12	11.34	2.98	3.46
GADD45A	39.94	40.31	27.13	14.01	13.68	13.80	1.37	0.97
GADD45B	71.42	72.19	80.44	13.16	12.24	11.83	2.59	3.45
GNG10	13.35	11.58	6.29	24.35	30.03	26.39	-1.37	-0.29
HDAC1	32.33	32.49	24.19	72.24	72.81	73.62	-1.30	-0.38
HLA-B	322.25	299.32	316.86	43.48	42.60	43.68	2.85	2.47
HLA-C	841.96	835.71	870.04	196.03	185.81	190.93	2.15	2.24
HLA-E	143.47	144.66	148.89	42.53	46.46	45.78	1.70	2.47
HSPA8	65.97	67.76	43.14	136.26	129.20	121.24	-1.13	0.06
JUNB	260.20	278.74	306.95	33.53	31.18	30.84	3.15	4.56
JUND	132.75	131.86	146.11	69.80	66.32	67.78	1.01	1.54
MAP2K3	30.37	31.04	31.07	14.75	11.93	11.90	1.26	2.03
MMP3	187.79	171.48	126.38	26.26	25.93	25.82	2.64	2.58
NFKB2	82.33	81.27	81.57	16.20	13.46	14.29	2.48	2.70
PCNA	33.06	34.85	20.69	80.48	80.95	74.55	-1.41	-0.56
PKMYT1	26.29	28.51	29.51	12.74	10.22	11.16	1.31	1.22
PPP2R5B	21.72	18.99	20.45	9.20	10.08	10.50	1.04	2.02
RELA	27.34	25.67	26.10	12.81	11.98	14.17	1.02	1.48
RIN1	22.23	22.46	23.80	12.38	10.36	11.23	1.01	1.75
RRAS	185.24	191.02	224.01	108.37	89.74	100.38	1.01	0.90
SERPINE1	1004.62	967.25	835.18	16.35	16.18	18.85	5.77	6.83
SESN2	47.94	47.16	48.20	16.62	16.71	17.17	1.50	1.92
SHC1	191.36	190.30	185.47	83.76	93.29	95.82	1.06	2.17
SPHK1	43.76	47.84	50.67	21.38	17.27	16.84	1.36	2.26
SQSTM1	85.02	86.13	85.87	43.96	42.07	41.82	1.01	0.91
SRF	42.54	41.35	42.29	19.76	21.79	21.02	1.01	1.42

Gene name	FPKM. NDV_1	FPKM. NDV_2	FPKM. NDV_3	FPKM. NC_1	FPKM. NC_2	FPKM. NC_3	log_2(FC) by RNA-seq	log_2(FC) by qPCR
STMN1	12.24	11.63	7.44	36.64	37.42	33.80	-1.78	-1.20
TP53I3	14.54	12.92	15.36	36.50	36.83	36.67	-1.36	-0.52
TUBA1A	50.50	46.66	49.30	111.66	109.36	109.96	-1.18	-0.28
VASP	169.14	164.88	168.78	73.69	68.38	73.80	1.22	1.52
VDAC3	15.30	13.48	8.60	34.98	34.34	33.44	-1.46	-0.58
YWHAQ	47.91	51.47	33.00	95.62	104.55	94.46	-1.15	-0.44
YWHAZ	21.78	21.49	12.67	42.99	49.03	45.46	-1.30	-0.16
ZYX	87.99	93.04	93.73	48.76	41.98	45.14	1.02	1.54
